# Health professionals' perspective on the promotion of e-mental health apps in the context of maternal depression

**DOI:** 10.1371/journal.pone.0180867

**Published:** 2017-07-12

**Authors:** Michaela Sprenger, Tobias Mettler, Jorge Osma

**Affiliations:** 1 University of St. Gallen, Institute of Information Management, St. Gallen, Switzerland; 2 University of Lausanne, Swiss Graduate School of Public Administration, Lausanne, Switzerland; 3 University of Zaragoza, Department of Psychology and Sociology, Teruel, Spain; Medizinische Universitat Wien, AUSTRIA

## Abstract

**Objective:**

Our study focuses on exploring (1) the intention of health professionals to use and recommend e-mental health applications, (2) how this intention of health professionals might be influenced, (3) which group of health professionals might be most accessible to promote e-mental health applications for maternal depression, and (4) for which tasks they rate them to be most useful.

**Materials and methods:**

Based on a questionnaire informed by the theory of planned behavior, we collected 131 responses of U.S., Spanish, and Swiss health professionals in the field of pregnancy and maternal care (including psychologists, psychiatrists, midwives, and doctors) by means of an online survey. We analyzed the gathered data applying a structured equation model.

**Results:**

Our study reveals that health professionals would in general intend to recommend and use e-mental health applications. However, their attitude towards e-mental health applications varies regarding the respective use cases and also differs among health professions.

**Conclusion:**

We offer three alternative propositions for private or public organizations, associations, or any other entity whose purpose is service to the community for introducing e-mental health applications into practice.

## Introduction

Being pregnant and becoming a mother is for most women a life-changing experience. However, not all women can cope with the new situation as emotional changes might cause maternal depression. This kind of depression includes all depressive conditions that occur either during pregnancy or within the first twelve months following delivery [1]. The percentage of women affected by maternal depression is significant: Depressions during pregnancy affect 38% [2] and 6 to 19.5%—depending on the country of reference—suffer from depressive conditions after giving birth [3, 4].

Women affected by maternal depression often feel hopeless or overstrained and might even not be able to cope with their role of being a mother [5]. Analogous to other depressive conditions, maternal depression is associated with a diminishing quality of life and a decrease in social functioning that negatively impacts not only the woman herself, but also the development of the (unborn) child [6, 7]. Especially with regard to the latter, maternal depression can lead to long-term consequences resulting in sub-optimal cognitive, behavioral, and emotional development of children [8] and adolescents [9]. Hence, the detection and treatment of maternal depression are important in order to prevent long-term consequences for women and their children. Despite this urgent necessity, one of the main problems is that maternal depression often remains unnoticed and consequently untreated as less than half of the cases are identified by health professionals in routine clinical practice [10, 11].

Possible reasons for this phenomenon have been identified, for example, the inadequate health professionals training about the problems related to maternal mental health, the low quality perception of the resources available, and the denial of depressive symptoms experienced by pregnant and postpartum women [12–14].

One of the proposed solutions to close the gaps mentioned above has been the use of e-mental health applications that provide mental health services via the Internet or related technologies [15] such as mental screening, psychological assessment, and treatment via ICT devices from anywhere, anytime, while usually saving time and money [16]. In addition, e-mental health applications could be good resources among health professionals, especially in primary care services, in order to help them promote mental health services in their routine practice [17]. In fact, practice models which allow a comprehensive incorporation of e-mental health resources into primary healthcare systems have recently been proposed [18].

In the field of maternal mental health, some relevant advantages of e-mental health applications for pregnant and postpartum women have been described. For example, since women can remain anonymous while using these applications they are more likely to share sensitive information regarding their mental health with reduced feelings of stigma [19, 20]. Moreover, an e-mental health application provides a low threshold to use which is especially true for mobile apps that allow for a non-intrusive utilization thanks to the ubiquity of smartphones in everyday life [21, 22]. As women can use the e-mental health application when it suits their time schedule, the flexibility is enhanced and the psychological consultation can be seen as a service on demand [19, 20]. From a societal perspective, e-mental health applications could not only increase the likelihood of detecting and treating maternal depression but also minimize costs as therapist contact time would be reduced and chances for a fast cure could be increased thanks to the early detection of the condition [19].

Due to these advantages, in recent years there has been a growing effort towards developing and testing e-mental health applications for maternal depression with positive outcomes [23–25] and high acceptability and feasibility ratings among users [20, 21, 26]. Despite all these advantages, yet their adoption and use is marginal. While existing studies dealing with user acceptance and use intention commonly focus on women as end-users, a possible explanation for low adoption rates could be related, first, to a limited number of evidence-based e-mental health applications available so far [32], and second, to the role and attitude of health professionals, among others.

**Fig 1:**
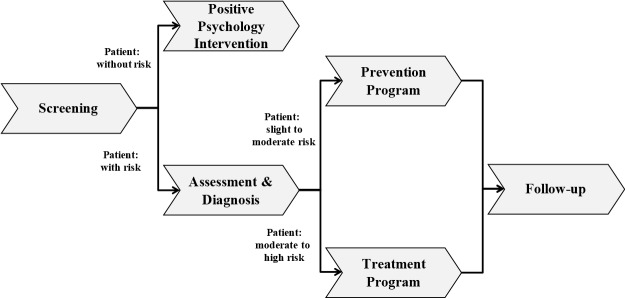
General mental healthcare process.

Our interest is focused on the role and attitude of health professionals as the adoption of e-mental health applications across the entire general mental healthcare process could be positively influenced by them [27–29]. This mental healthcare process includes the general steps that are at the heart of the science of psychology and applicable to a wide variety of mental health issues [30]. Fig 1 gives an illustrative overview: The first step of the general mental healthcare process is screening. Screening refers to an initial patient evaluation including medical and psychiatric history, mental status, as well as the patient’s suitability for a particular treatment modality [31]. If the screening outcomes show no risk tendencies, people can benefit from a positive psychology intervention in order to increase their psychological resources to cope with daily stress [32–34]. If the screening indicates the patient to be at risk of a psychological condition, he will be thoroughly assessed as well as diagnosed and—depending on the identified level of risk—transferred to a prevention [35, 36] or treatment program [37]. It is recommended to conduct a follow-up assessment over time in order to verify that the prevention and treatment programs have the envisioned long-term results [38].

Several studies have shown that the successful implementation of digitized services within healthcare not only requires the integration of work practices and processes [39, 40], timely as well as role-specific exchange of information and education [41, 42], but also in most cases effective personal interaction between the different actors involved [43, 44]. For instance, according to the Consolidated Framework for Implementation Research (CFIR) [45] the consideration of the inner and outer setting as well as the needs and requirements of affected individuals are important antecedents for a successful implementation of new (digitized) processes and interventions. In this sense, the integration of diverse perspectives prior to deciding about the implementation of new digitized services is crucial and helps to identify potential obstacles and opportunities from introducing new technological solutions in an early stage of the process [46]. While there has been a strong emphasis in identifying these obstacles and opportunities in clinical medicine, only sparse knowledge exists about the expectations, actual needs, and attitudes of health professionals, who work in the area of clinical and health psychology [47]. Since the recommendation and guidance by trustworthy coaches, such as therapists, have a positive influence on the usage of e-mental health applications [27, 48, 49], the perspective of healthcare providers has to be considered when studying the potential use of e-mental health applications in the context of maternal depression [22]. For this reason, the aim of this study is to figure out if health professionals working with pregnant and postpartum women would act as promoters for e-mental health applications to prevent maternal depression [18].

## Materials and methods

### Research model

**Fig 2:**
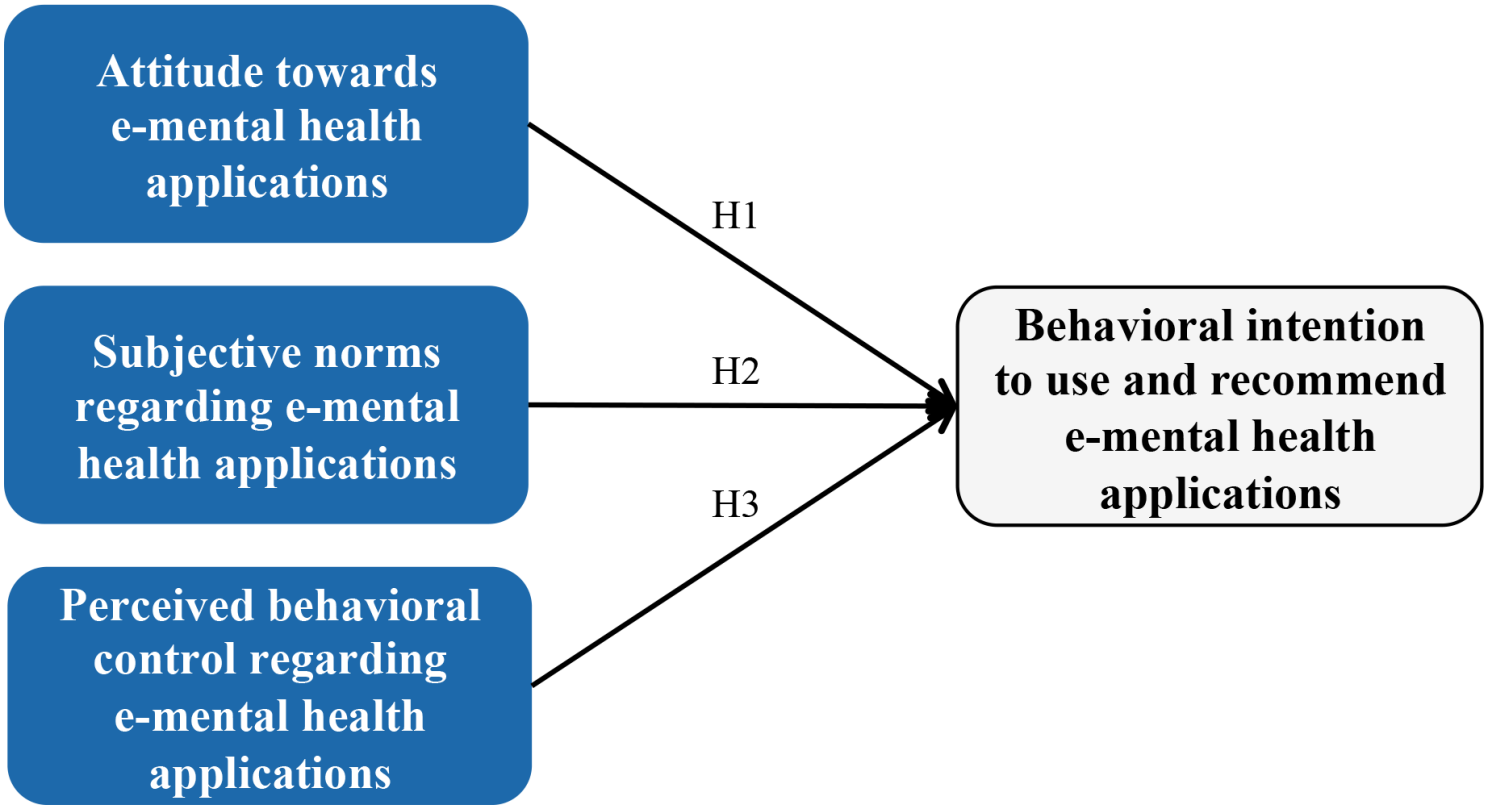
Research model.

Our study concentrates on analyzing the behavioral intention of health professionals towards using and recommending e-mental health applications for maternal depression. To investigate behavioral intentions of health professionals, we base our work on the theory of planned behavior (TPB) [50]. TPB uses three constructs: attitude, subjective norms, and perceived behavioral control, which we will contextualize for this study and explain next (Fig 2).

The attitude reflects in our case the degree to which a health professional has a favorable or unfavorable appraisal regarding the use and recommendation of e-mental health applications in the context of maternal depression [51]. According to the theory of planned behavior, the constructs “attitude” and “behavioral intention” correlate positively with each other. Hence, we derive:

***Hypothesis 1**: Positive attitudes increase the behavioral intention to use and recommend e-mental health applications in the context of maternal depression*.

Subjective norms refer to the health professionals’ believes about what their colleagues or other important people in their lives might recommend them to do [52]: Would they suggest them to use and recommend e-mental health applications for maternal depression? Based on the theory of planned behavior, a positive correlation between the construct “subjective norms” and “behavioral intention” is to be expected. Thus, we suggest:

***Hypothesis 2**: Subjective norms that favor using and recommending e-mental health applications in the context of maternal depression will have a positive effect on the corresponding health professional’s behavioral intention*.

In our case, perceived behavioral control reflects to which extent health professionals think that it is easy or difficult to use and recommend e-mental health applications for maternal depression [51]. In line with the theory of planned behavior, a positive correlation between the constructs “perceived behavioral control” and “behavioral intention” can be anticipated. Therefore, we propose:

***Hypothesis 3**: Higher levels of perceived behavioral control will have a positive effect on the behavioral intention to use and recommend e-mental health applications in the context of maternal depression*.

With regard to the operationalization of constructs we strived for reuse and adaptation of existing measurement items in the literature. As a plethora of studies already applied the theory of planned behavior [53], we searched the literature to identify reusable items that already measured the constructs attitude, subjective norms, perceived behavioral control, and behavioral intention. Based on [54], we adjusted the identified measurement items to our research interest by adapting them to the context of e-mental health applications for maternal depression. For example, [55] applied the item “Using telemedicine technology would be entirely within my control” as one operationalization for the construct “perceived behavioral control” in their telemedicine study. We transferred this operationalization in our study to “Recommending and using e-mental health applications in the context of maternal depression would be totally in my control”. Analogously, we developed the items for all constructs by reusing and adapting measurement items of other TPB studies presented in literature. In line with [55–57], we developed three items per construct that will be discussed later.

### Research method and data analysis

To test our hypotheses and to identify the health professionals’ behavioral intention, we conducted a survey that was based on an online questionnaire. The participants of the survey were asked to indicate their level of agreement with the statements used as measurement items for our constructs on a Likert scale from 1 (strongly disagree) to 5 (strongly agree).

As our research interest was not only to determine the behavioral intention and the influencing factors, but also to identify the steps in the general mental healthcare process where an e-mental health application would be most useful, we included a corresponding question in our survey. Moreover, we asked additional questions regarding profession, the role of technology in the work setting, as well as the expertise in the field of maternal depression. These questions were intended to identify differences among groups (cf. S1 File for an overview of the survey questions).

The link to the online survey was distributed in Europe and the United States of America via email and online platforms targeting health professionals in the field of pregnancy related work. Overall, we received 131 answers which were all complete as the online tool required the participants to answer each question before they could proceed to the next one and only saved the answers after asking permission for it at the very end of the survey. On average, the health professionals were 46 years old and practicing their job for 19 years. The participants were mostly midwives and nurses involved in maternal care (79), psychologists and psychiatrists (27), as well as doctors (12), whereas the remaining answers came from other health professions (e.g. health professionals at research institutes). We deem this distribution to be suitable as most pregnant women and new mothers spend a lot of time with midwifes and nurses (at the doctor’s office during pregnancy, in the hospital during childbirth, and at home during house calls after giving birth). Psychologists and psychiatrists are an important group as they deal with all kinds of mental health issues, including depressions. The opinions of doctors involved in the maternal process are also important as they regularly consult with their female patients during pregnancy and after giving birth. To ensure that our results are not biased, we used tests that accounted for the unequal distribution of professions among the survey participants when performing statistical analyses regarding differences among professions.

For data analysis, we transferred our research model into a structural equation model with reflective measurements to test it with the help of a partial least squares (PLS) approach. Here, we applied the SmartPLS2.0.M3 software package [58]. Following [59], this approach has advantages over linear regression models as it allows the simultaneous analysis of the relationship between latent variables (i.e. our constructs) and their respective indicators (i.e. our measurement items). Moreover, in contrast to covariance-based structural equation models (CBSEM), PLS focuses rather on the predictive power of the model than on its accuracy [60]. In our case, this is suitable as we apply a model that already proved to be accurate in various studies [61].

The structural equation model requires, in general, a smaller sample size compared to other approaches like CBSEM [62]. In [63] an example of a research model is presented where the maximum number of independent variables in the measurement and structural model is five: Here, a sample size of 70 would be needed to reach a statistical power of 80% with rather small effect sizes (i.e. 0.25) at a significance level of 5%. For our research model (with a maximum number of three independent variables in the measurement and structural model) this approach suggests a sample size between 30 and 124 –depending on the effect sizes—with a significance level of 5%. The fact that our sample size is sufficient is corroborated by the quality criteria and the significant levels of our results, which we will elaborate on in the following. The significances of all estimates were verified by two-tailored t-test, whereas we applied the bootstrapping function within SmartPLS with 500 resamples as suggested by [64].

The results of the PLS approach are evaluated according to standardized reporting requirements [59].

For the measurement model these requirements include (i) internal consistency, (ii) indicator reliability, (iii) convergent validity, and (iv) discriminant validity [63]:

Internal consistency is often measured with Cronbach’s alpha (CA) which represents the inter-correlation of the measurement items. CA is based on the assumption that a construct’s measurement items have equal loadings. As this assumption is not always met, it is advisable to additionally calculate the composite reliability (CR) which accounts for differences between the measurement item’s loadings. For both CA and CR, values should be above 0.7, whereas our results meet these quality criteria (Table 1).

**Table 1:** Latent variables’ correlations, square root of AVE on main diagonal, and quality criteria.

	*Attitude*	*Subjective norms*	*Perceived behavioral control*	*Behavioral intention*	*AVE*	*CA*	*CR*
*Attitude*	0.94				0.89	0.94	0.96
*Subjective norms*	0.49	0.96			0.92	0.96	0.97
*Perceived behavioral control*	0.65	0.40	0.83		0.69	0.78	0.87
*Behavioral intention*	0.79	0.45	0.76	0.83	0.69	0.76	0.86

Indicator reliability reflects how much of an item’s variation is explained by its construct. This quality criterion is met when the loadings of the measurement items (λ) are above 0.7. This is true for all but one of our measurement items at a significance level of α<0.01 (cf. Table 2). The deletion of a measurement item that does not meet the required value of 0.7 is usually recommended, if the CA and CR values of the respective construct do not meet the quality criteria and could be improved by excluding the measurement item. In our case, the CA and CR values for the construct “behavioral intention” are already meeting the quality criteria. Hence, the measurement item “behavioral intention_2” will be included in the model even though its loading has only a value of 0.6.

**Table 2:** Measurement model (incl. mean value μ, standard deviation σ, and factor loading λ).

*Item*	*Description*	*μ*	*σ*	λ***
*Attitude_1*	In terms of cost-benefit, it is beneficial to use e-mental health applications in the context of maternal depression.	3.60	1.17	0.93
*Attitude_2*	E-mental health applications would be useful in the context of maternal depression.	3.65	1.22	0.95
*Attitude_3*	Using e-mental health applications in the context of maternal depression is in general a good idea.	3.66	1.20	0.96
*Subjective norms_1*	People who influence my clinical behavior think that I should recommend and use e-mental health applications in the context of maternal depression.	2.79	1.31	0.94
*Subjective norms_2*	People who are important in the selection of my healthcare services think that I should recommend and use e-mental health applications in the context of maternal depression.	2.90	1.29	0.97
*Subjective norms_3*	People who are important in assessing my patient care and management think that I should recommend and use e-mental health applications in the context of maternal depression.	2.95	1.25	0.97
*Perceived behavioral control_1*	I would have the ability to recommend and use e-mental health applications in the context of maternal depression.	3.56	1.33	0.91
*Perceived behavioral control_2*	Recommending and using e-mental health applications in the context of maternal depression would be totally in my control.	3.34	1.30	0.84
*Perceived behavioral control_3*	I would have the knowledge to recommend and use e-mental health applications in the context of maternal depression.	3.02	1.41	0.74
*Behavioral intention_1*	I would be willing to inform pregnant women / new mothers I attend of e-mental health applications and their utility.	3.78	1.35	0.90
*Behavioral intention_2*	Whenever possible I intend to recommend and use e-mental health applications in the context of maternal depression.	2.90	1.39	0.60
*Behavioral intention_3*	I would recommend and use e-mental health applications in the context of maternal depression.	3.47	1.36	0.95

Note:

***All factor loadings are significant with α<0.01

In addition to indicator reliability, the average variance extracted (AVE) is analyzed to see if convergent validity is given. Whereas the indicator reliability reflects the communality of an item, the AVE can be referred to as the communality of a construct. The AVE is determined by calculating the mean value of the construct’s squared item loadings and should be above 0.5. In our case, this condition is met for all our constructs (Table 1).

Discriminant validity deals with the question how much the constructs differ from each other. This can be evaluated by the Fornell-Larcker criterion that requires the construct’s square root of the AVE to be greater than its correlations with the other constructs. As this is true for all our constructs, our measurement model meets the discriminant validity criterion.

For data analysis regarding differences among professions we used the statistical software IBM SPSS Statistics 21 [65]. As we tested for the differences among three groups (i.e. midwifes and nurses involved in maternal care (n = 79), psychologists and psychiatrists (n = 27), as well as doctors (n = 12)), we applied a one-way ANOVA to check if there are significant differences among professions and a post-hoc Bonferroni test to detect among which groups these differences are present.

## Results

### Health professionals’ behavioral intention

**Fig 3:**
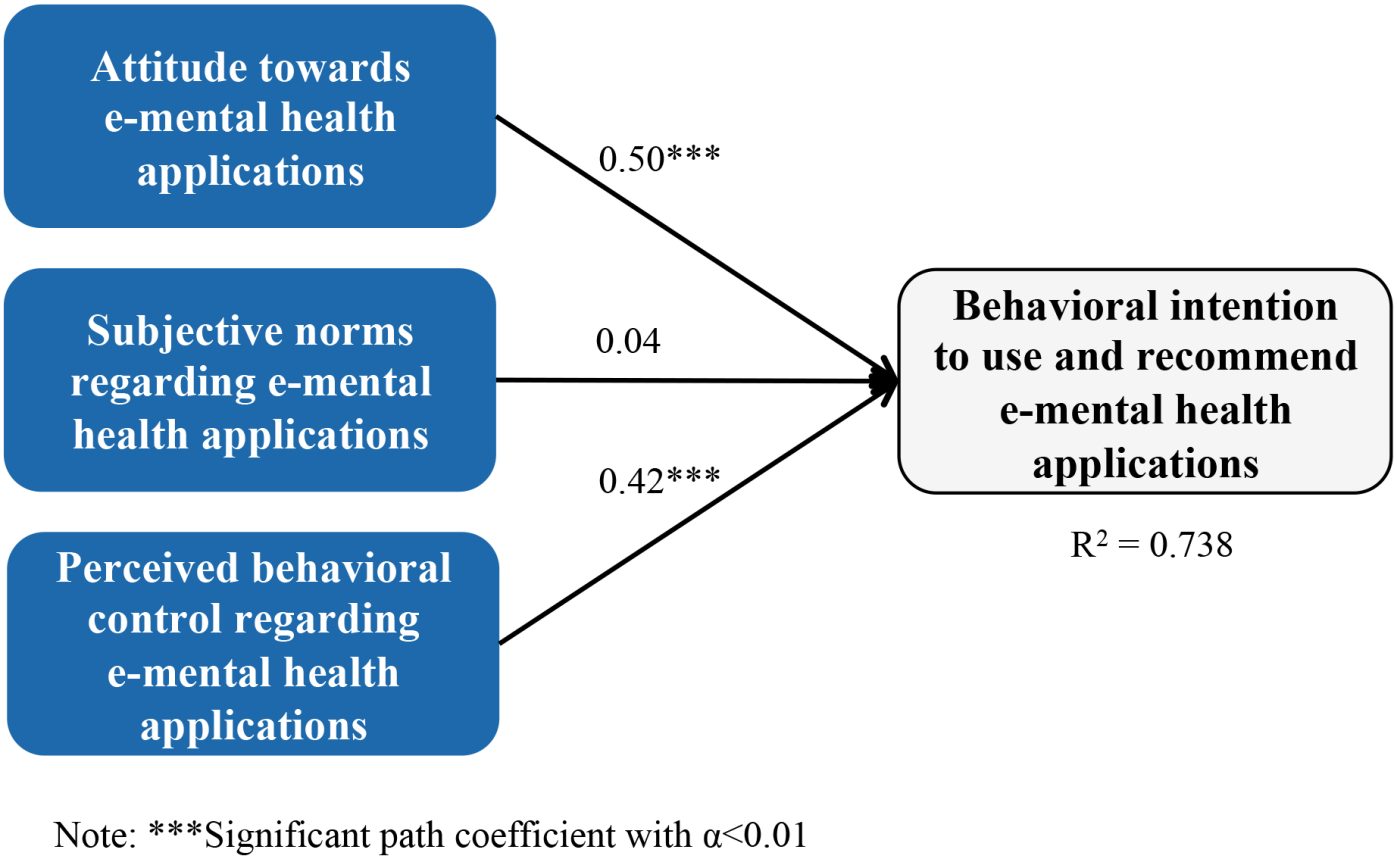
Structural model results.

The significance and variance of path estimates regarding the health professionals’ behavioral intention to use and recommend e-mental health apps is explained by the structural model [60], which we will present next. As expected by the hypotheses, all path coefficients are positive. However, only the constructs “attitude” and “perceived behavioral control” are highly significant (Fig 3). The high value of R^2^ (0.738) shows that the model is able to explain a high level of variance regarding the construct “behavioral intention”. Additionally, we calculated the Stone-Geisser value Q^2^ which, in case Q^2^>0, indicates that the model has predictive power [63]. In our case the Q^2^ value is 0.4988, meaning the behavioral intention to use and recommend e-mental health applications in the context of maternal depression can be predicted by the exogenous constructs (i.e. attitude, subjective norms, and perceived behavioral control).

After the measurement model and the structural model are evaluated, the results can be analyzed from a content perspective. Looking at the mean values of the independent latent variables’ measurement items (Table 2) our results reveal that health professionals have a positive attitude towards e-mental health applications in the context of maternal depression (all measurement items’ mean values of “attitude” >3) and that they perceive their corresponding behavioral control to be rather high (all measurement items’ mean values of “perceived behavioral control” >3). As the values for the construct “subjective norms” are below average (all measurement items’ mean values of “subjective norms” <3), the influence of the health professionals’ environment to use and recommend e-mental health applications in the context of maternal depression is rather low. With regard to our hypotheses, our research results corroborate that the behavioral intention to use and recommend e-mental health applications for maternal depression depends on the attitude towards these applications (hypothesis 1) and the perceived behavioral control to use and recommend them (hypothesis 3): The more favorable the attitude and the higher the perceived behavioral control, the higher the behavioral intention to use and recommend e-mental health applications for maternal depression. Considering the high mean values of the measurement items for the constructs “attitude” and “perceived behavioral control” as well as the significant, positive influence of these two constructs on the behavioral intention, it is no surprise that the mean values of the dependent latent variable are rather high (two of the three measurement items’ mean values of “behavioral intention” >3). Hence, health professionals seem to be willing to inform pregnant women and new mothers about the utility of these e-mental health applications. They would also use and recommend them, but not whenever possible (mean value of “behavioral intention_2” <3).

As health professionals would not use and recommend e-mental health applications in the context of maternal depression whenever possible, we will further analyze the perceived usefulness of such an application for the different steps of the aforementioned general mental healthcare process. The idea is to identify those process steps for which it would be most beneficial to support them with e-mental health applications. Moreover, we will analyze if there are differences among different health professions in order to detect the group of health professionals that would be most accessible regarding e-mental health applications for maternal depression.

### Utility of e-mental health applications depending on mental healthcare process steps and health professions

To determine for which steps of the general mental healthcare process e-mental health applications could be most useful, the mean values per process step can be consulted (1: not at all useful, 5: extremely useful). As all mean values are >3 (Table 3), health professionals seem to see the value of such applications for each process step. With regard to the process steps “screening” and “prevention program”, e-mental health applications are judged to be especially useful. As our research interest is also in detecting potential differences among health professions, the mean values of the three different groups, i.e. doctors, midwifes / nurses, and psychologists / psychiatrists, have to be compared. In order to evaluate if the values are significantly different from each other, a one-way ANOVA has to be calculated followed by a Bonferroni post-hoc test that indicates between which groups these significant differences are present.

**Table 3:** Usefulness of e-mental health applications for maternal depression per process step and health profession.

*How useful would you find e-mental health applications regarding maternal depression for the following steps of the general mental healthcare process (1*: *not at all useful*, *5*: *extremely useful)*?	*Doctors*	*Midwifes / Nurses*	*Psychologists / Psychiatrists*	*Overall*
Screening	3.83	4.15	3.67	**3.88**
Positive psychology intervention*	3.50	3.73	3.07	**3.43**
Assessment and diagnosis**	3.67	3.49	2.74	**3.30**
Prevention program**	4.00	4.14	3.26	**3.80**
Treatment program**	3.67	3.47	2.78	**3.31**
Follow-up	3.75	3.84	3.56	**3.72**
Overall**	**3.74**	**3.80**	**3.18**	**3.57**

Note:

*Significant differences among professions with α<0.1, psychologists / psychiatrists being significantly different form the other groups;

**Significant differences among professions with α<0.05, psychologists / psychiatrists being significantly different form the other groups

Our results show that there are significant differences among professions regarding the following general mental healthcare process steps: positive psychology intervention, assessment and diagnosis, prevention program, and treatment program (Table 3). The statistical test reveals that these significant differences exist between psychologists / psychiatrists and the other groups (i.e. doctors and midwifes / nurses). Although psychologists / psychiatrists see the usefulness of e-mental health applications in the context of maternal depression for most steps in the general mental healthcare process, they are rather skeptical when it comes to the assessment and diagnosis as well as the treatment of the maternal depression (mean values are <3).

As extant literature concerning adoption barriers of e-mental health identified the lack of technology usage and the fear of being replaced by technology as one of the main reasons why psychologists / psychiatrists are skeptical concerning e-mental health applications [66, 67], we analyzed the responses to the survey questions regarding the use of technology in daily work and the expertise in the field of maternal depression. Here, we hoped to find possible answers, even though there might be other reasons why psychologists / psychiatrists are more hesitant to use and recommend e-mental health applications for maternal depression (which we will further elaborate on in the Discussion section).

The role of technology in the work setting was analyzed with the help of three questions (Table 4; upper part), whereas the survey included a definition and an example for clarification. The results show that technology is beneficial for all health professions (all values are >3). However, the comparison between groups reveals differences between the group of psychologists / psychiatrists and the two other groups, whereas the values of psychologists / psychiatrists are significantly lower. Hence, psychologists / psychiatrists do not use technology for their work as often as other health professionals do and they do not judge technology to be that useful or beneficial for their work compared to doctors and midwifes / nurses.

**Table 4:** Role of technology and expert knowledge of surveyed health professionals.

*1*: *not at all*, *5*: *extremely (often/useful)*	*Doctors*	*Midwifes / Nurses*	*Psychologists / Psychiatrists*	***Overall***
How often do you use technology for your work?***	4.33	4.22	3.30	**3.95**
How useful is technology for your work?***	4.58	4.51	3.48	**4.19**
How much does technology improve your work?***	4.42	4.05	3.37	**3.95**
*1*: *no knowledge*, *5*: *substantial knowledge*	*Doctors*	*Midwifes / Nurses*	*Psychologists / Psychiatrists*	***Overall***
Do you have knowledge about the symptomatology of maternal depression?***	3.58	3.75	4.85	**4.06**
Do you have knowledge of the factors that increase the risk of maternal depression?***	3.42	3.57	4.89	**3.96**

Note:

***Significant differences among professions with α<0.01, psychologists / psychiatrists being significantly different form the other groups

Concerning the threat of being replaced by technology, we analyzed if psychologists / psychiatrists have a significantly higher level of knowledge regarding maternal depression as this would reflect their expertise in the field and indicate a potential rivalry between their job and the e-mental health applications. Per definition, the diagnosis and treatment of mental health conditions is at the heart of their job. This would also explain why they find e-mental health applications most useful for screening (first process step) and follow-up (last process step), whereas they judge the applications to be less useful for the core steps of the general mental healthcare process. The results confirm that psychologists / psychiatrists have a significantly higher level of knowledge concerning maternal depression (Table 4; lower part).

## Discussion

**Fig 4:**
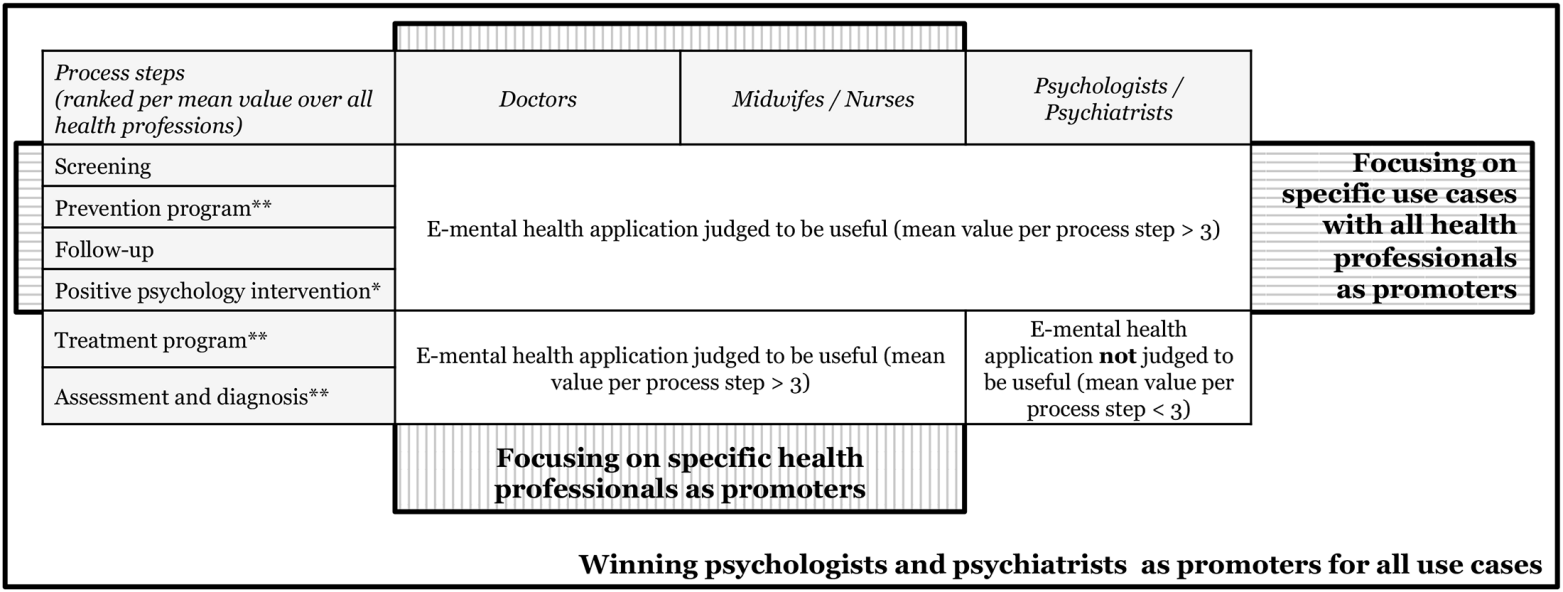
Options for introducing e-mental health applications in practice.

Overall, our study has important implications for research and practice. From an academic perspective, we conducted—to our knowledge—the first survey that concentrated on the different groups of health professionals involved in maternal treatment and care (doctors, midwifes, nurses, psychologists, psychiatrists) and on their perspective towards e-mental health applications in the context of maternal depression. By applying the theory of planned behavior, our study provides insights on the behavioral intention of health professionals and on the underlying factors. Moreover, we evaluated the perceived usefulness of the application per mental healthcare process step and per health profession. Thereby, our study goes one level deeper by not only focusing on behavioral intention overall, but by also giving insights on which process step(s) e-mental health applications are judged to be of most value for and which groups of health professionals are most accessible for promoting these applications. In view of the usefulness of e-mental health applications per process step and health professional group, the above presented results show that there are differences between distinct professional groups. Overall, psychologists and psychiatrists attribute a lower level of usefulness to each process step compared to the other surveyed health professionals. Still, they judge e-mental health applications for maternal depression as useful for screening, prevention program, follow-up, and positive psychology intervention. In contrast to the other health professions, they do not value e-mental health applications for the treatment or assessment and diagnosis of maternal depression. When it comes to the question how e-mental health applications could be promoted, we would like to offer three propositions (Fig 4), which we describe next.

### Focusing on specific use cases

When focusing on specific use cases, our results would suggest an application that supports screening, prevention program, follow-up, and positive psychology intervention as all health professions see here the most value. Since the attitude has the highest impact on the behavioral intention to use and recommend the e-mental health application, a provider of such an application (i.e. a private or public organization, association, or any other entity whose purpose is service to the community) would have to clearly define the value propositions for all use cases in order to convince the health professionals of the application’s value. Often, the value of e-mental health is presented in such a way that it addresses only patients (e.g. e-mental health as a way to improve the convenience and accessibility) or health systems (e.g. e-mental health as means of cost reduction for the overall health system) [66]. To ensure that health professionals promote an e-mental health application for maternal depression, the value proposition has also to exhibit the benefits for them. For example, in the case of “screening”, a provider of an e-mental health application for maternal depression could highlight the application’s utility for a first assessment which ensures that women that are at risk of maternal depression are motivated to contact a health professional. For women with no risk, the application would rather create awareness for the topic of maternal depression itself. Thereby the health professionals’ efficiency might be increased, as the application helps them to focus rather on women that are really at risk.

When opting for addressing only specific use cases, the provider of an e-mental health application would also need to define the interfaces between the application-supported process steps and the ones that are not supported by the application. For example, if the application would support screening but not the assessment and diagnosis step, the application could recommend the women to go to a health professional for a thorough assessment in case the screening would indicate a certain risk for maternal depression.

Even though focusing on specific use cases is a valid approach, the downside is that the non-supported process steps are left out although the application might also be beneficial for them. Extant studies revealed the (potential) value of e-mental health applications for the treatment as well as the assessment and diagnosis both in the context of other mental health conditions [68–71] and related to maternal depression [21].

### Focusing on specific health professionals

In order to leverage the potential benefits for the entire mental healthcare process, another option would be to address all process steps with the application, but to focus only on doctors as well as midwifes and nurses to promote the adoption of an e-mental health application. Since more pregnant women and new mothers are rather in contact with a doctor or a midwife / nurse than with a psychologist / psychiatrist, focusing on the former health professions might be a suitable option. Moreover, psychologists / psychiatrists have probably more patients with severe depression where they might not see technology support as an option at all [67]. Similar to focusing on specific use cases, an application provider would need to define a compelling value proposition for doctors as well as midwifes and nurses. As these health professionals might be rather confronted with not yet diagnosed or mild depression cases, the application could aim at supporting them here.

The disadvantage of only concentrating on specific health professions other than psychologists and psychiatrists is that the potential value of an e-mental health application for rather severe depression candidates is not leveraged. Even though these cases might need more face to face therapy time, an e-mental health application could still be useful to complement the conventional therapeutic service [72].

### Winning psychologists and psychiatrists for all use cases

The third option would be to target all health professionals for all use cases with the e-mental health application. However, a prerequisite would be to understand first, why psychologists / psychiatrists do not perceive an e-mental health application to be useful for the treatment or assessment and diagnosis of maternal depression. Extant literature as well as our results suggest that technology in general is not seen as important in the daily work for psychologists / psychiatrists as for other health professions. Demonstrating the advantages for the psychologists and psychiatrists of an e-mental health application is therefore crucial. For example, an e-mental health application could be used for a first assessment and diagnosis, especially for those women who would refrain from seeking help by a health professional. In case the application would identify a woman to have a high risk of depression, the application might encourage her to go see a psychologist or psychiatrist [22]. Thereby, the application would give health professionals access to new clients and thereby to an additional source of revenue. By stressing the advantages for the health professionals, psychologists and psychiatrists might be less inclined to see e-mental health applications as a form of competition, which was suggested by extant research as well as our results to be another potential reason for their rather skeptical attitude. To get their support, an e-mental health application for maternal depression would have to be positioned as a beneficial, complementary service and not as a substitute to psychological consultations. By clarifying this, the attitude as well as the perceived behavioral control of psychologists / psychiatrists towards an e-mental health application could be enhanced which would have a positive effect on their behavioral intention to use and recommend these applications and become valuable promoters.

## Conclusion and limitations

The insights presented in this paper are relevant from a practical point of view. Service providers aiming at developing an e-mental health application for maternal depression can use the results of our paper to inform the design of their service (e.g. which steps of the general mental healthcare process should be covered by the application) as well as the corresponding go-to-market approach (e.g. which group of health professionals would support the application; how could other groups be convinced of its value).

Still, our study is not without limitations. Our investigation has focused primarily on analyzing the expectations, actual needs, and attitudes of health professionals. In this sense, our results portray the “soft” or human side, concentrating only on one of the five major elements of implementation research. Hence, future research could—as suggested by the CFIR [45]–extend our work by exploring additional nuances regarding the intervention, inner and outer setting, and implementation process itself.

Furthermore, although we tried to cover all groups of health professionals that are relevant in our research setting, we cannot judge the representativeness of our sample. As this is especially true concerning the number of participants per group, we opted for statistical tests that accounted for the different group sizes when analyzing differences among health professions. Another important limitation is that we have no guarantee that a positive behavioral intention will lead to a successful introduction and adoption of an e-mental health application for maternal depression. Besides the technical feasibility and the positive evaluation of relevant stakeholders, such an e-mental health application has to be economically viable in order to survive in the long run [73]. Future research in this area could shed some light on suitable business models, especially regarding the identification of adequate revenue mechanisms as well as the stakeholders that would be willing to pay them.

## Supporting information

S1 File Questionnaire used for data collection in this study. (PDF)

S1 Dataset Complete data set for the analyses presented in this study. (CSV)
