# Supplementary material for: Health professionals' perspective on the promotion of e-mental health apps in the context of maternal depression
Source: PLoS One. 2017 Jul 12;12(7):e0180867. doi: 10.1371/journal.pone.0180867 (PMC5507525; doi:10.1371/journal.pone.0180867)
Supplement: S1 File — (PDF) [file pone.0180867.s001.pdf]

S1 Appendix – Questionnaire

Demographics

Age __________

Job title __________

Years of practicing __________

Role of technology in your work setting

Please answer the following questions on a scale from 1 (not at all) to 5 (extremely often/useful)

	Not at all (1) – Extremely often/useful (5)
How often do you use technology for your work?	①②③④⑤
How useful is technology for your work?	①②③④⑤
How much does technology improve your work?	①②③④⑤

Knowledge regarding maternal depression

Please answer the following questions on a scale from 1 (no knowledge) to 5 (substantial knowledge)

	No knowledge (1) – Substantial knowledge (5)
Do you have knowledge about the symptomatology of maternal depression?	①②③④⑤
Do you have knowledge of the factors that increase the risk of maternal depression?	①②③④⑤

Questions regarding an e-mental health app for maternal depression

On a scale from 1 (strongly disagree) to 5 (strongly agree), what is your level of agreement with the following statements?

Statements	Strongly disagree (1) - Strongly agree (5)
In terms of cost-benefit, it is beneficial to use e-mental health applications in the context of maternal depression.	①②③④⑤
E-mental health applications would be useful in the context of maternal depression.	①②③④⑤
Using e-mental health applications in the context of maternal depression is in general a good idea.	①②③④⑤

People who influence my clinical behavior think that I should recommend and use e-mental health applications in the context of maternal depression.	①②③④⑤
People who are important in the selection of my healthcare services think that I should recommend and use e-mental health applications in the context of maternal depression.	①②③④⑤
People who are important in assessing my patient care and management think that I should recommend and use e-mental health applications in the context of maternal depression.	①②③④⑤
I would have the ability to recommend and use e-mental health applications in the context of maternal depression.	①②③④⑤
Recommending and using e-mental health applications in the context of maternal depression would be totally in my control.	①②③④⑤
I would have the knowledge to recommend and use e-mental health applications in the context of maternal depression.	①②③④⑤
I would be willing to inform pregnant women / new mothers I attend of e-mental health applications and their utility.	①②③④⑤
Whenever possible I intend to recommend and use e-mental health applications in the context of maternal depression.	①②③④⑤
I would recommend and use e-mental health applications in the context of maternal depression.	①②③④⑤

Based on the presented general mental healthcare process, how useful would you find e-mental health applications regarding maternal depression for the following steps of this process?

Steps of the general mental healthcare process	Not at all useful (1) – extremely useful (5)
Screening	①②③④⑤
Positive psychology intervention	①②③④⑤
Assessment and diagnosis	①②③④⑤
Prevention program	①②③④⑤
Treatment program	①②③④⑤
Follow-up	①②③④⑤
